# CSmiRTar: Condition-Specific microRNA targets database

**DOI:** 10.1371/journal.pone.0181231

**Published:** 2017-07-13

**Authors:** Wei-Sheng Wu, Bor-Wen Tu, Tsung-Te Chen, Shang-Wei Hou, Joseph T. Tseng

**Affiliations:** 1 Department of Electrical Engineering, National Cheng Kung University, Tainan, Taiwan; 2 Department of Biotechnology and Bioindustry Sciences, National Cheng Kung University, Tainan, Taiwan; University College London, UNITED KINGDOM

## Abstract

MicroRNAs (miRNAs) are functional RNA molecules which play important roles in the post-transcriptional regulation. miRNAs regulate their target genes by repressing translation or inducing degradation of the target genes’ mRNAs. Many databases have been constructed to provide computationally predicted miRNA targets. However, they cannot provide the miRNA targets expressed in a specific tissue and related to a specific disease at the same time. Moreover, they cannot provide the common targets of multiple miRNAs and the common miRNAs of multiple genes at the same time. To solve these two problems, we construct a database called CSmiRTar (Condition-Specific miRNA Targets). CSmiRTar collects computationally predicted targets of 2588 human miRNAs and 1945 mouse miRNAs from four most widely used miRNA target prediction databases (miRDB, TargetScan, microRNA.org and DIANA-microT) and implements functional filters which allows users to search (i) a miRNA’s targets expressed in a specific tissue or/and related to a specific disease, (ii) multiple miRNAs’ common targets expressed in a specific tissue or/and related to a specific disease, (iii) a gene’s miRNAs related to a specific disease, and (iv) multiple genes’ common miRNAs related to a specific disease. We believe that CSmiRTar will be a useful database for biologists to study the molecular mechanisms of post-transcriptional regulation in human or mouse. CSmiRTar is available at http://cosbi.ee.ncku.edu.tw/CSmiRTar/ or http://cosbi4.ee.ncku.edu.tw/CSmiRTar/.

## Introduction

MicroRNAs (miRNAs), 20–25 nucleotides non-coding RNAs, play important roles in the post-transcriptional regulation of gene expression [1–3]. Via binding to the complementary sites within the 3’ untranslated regions (3’ UTRs) of their target genes’ mRNAs, miRNAs induce mRNA degradation or lead to translational inhibition [1,4–6]. miRNAs are known to be involved in a wide range of biological processes including cell development, differentiation, cell-cycle control and apoptosis [7–9].

Understanding the miRNA-target interactions is the crucial step to discern the roles of miRNAs in different biological processes [10]. Many databases have been constructed to provide miRNA targets information. For example, TarBase [11] and miRTarBase [12] collect manually curated miRNA targets with experimental evidence from the literature but they are far from complete. The other databases such as TargetScan [10], miRDB [13], microRNA.org [14], DIANA-microT [15], miRecords [16], MAGIA [17], mirDIP [18], miRSystem [19] and miRGator [20] collect computationally predicted miRNA targets generated from various algorithms. However, these databases usually return thousands of predicted targets of a query miRNA. Researchers have to put extra efforts to extract the interested miRNA targets from a large number of uninterested ones. Since miRNAs regulate their targets in specific tissues, cell types and disease states, it is advantageous to have a database which can return miRNA targets in a specific physiological condition. Three existing databases attempted to meet this need. miTALOS [21] can provide miRNA targets of a specific tissue or cell line. miRWalk [22] can provide miRNA targets related to a specific OMIM disorder. starBase [23] can provide miRNA targets whose expressions are anti-correlated with miRNA’s expression in specific cancer types. However, none of them can provide the miRNA targets expressed in a specific tissue and related to a specific disease at the same time. Therefore, there is still a need for a database which implements both the tissue and disease filters.

The complex circuitry of miRNA-mRNA interactions show the dynamic regulation of gene expression. Recent study showed that overexpressed MRE (miRNA response element)-containing transcripts can soak up the miRNA and upregulate its target genes [24]. Moreover, the competing endogenous RNAs (ceRNAs), transcripts that cross-regulate each other by competing for shared miRNAs, were proposed to describe the new layer of post-transcriptional regulation and linked the functions of coding and non-coding RNAs [25]. Several studies indicated the deregulation of ceRNA network in cancer development [26]. Because most existing databases do not provide the common miRNAs of a set of genes, they cannot be used to find out the shared miRNAs of ceRNAs. Therefore, it is advantageous to have a database which provides researchers the common miRNAs of multiple genes and the common targets of multiple miRNAs.

To meet these two needs, we develop a database called CSmiRTar (Condition-Specific miRNA Targets). CSmiRTar collects computationally predicted targets of 2588 human miRNAs and 1945 mouse miRNAs from four most widely used miRNA target prediction databases (miRDB, TargetScan, microRNA.org and DIANA-microT). CSmiRTar implements (i) a tissue filter for users to search the miRNA targets expressed in a specific tissue, (ii) a disease filter for users to search the miRNA targets related to a specific disease, and (iii) a database filter for users to search the miRNA targets supported by multiple existing miRNA target prediction databases. Moreover, CSmiRTar allows users to search the common targets of a set of input miRNAs under a specific physiological condition and the common miRNAs of a set of input genes under a specific physiological condition. We believe that CSmiRTar will be a useful database for biologists to study the molecular mechanisms of post-transcriptional regulation in human or mouse.

## Construction and contents

### Data collection and processing

Five data sources were used to construct CSmiRTar. First, the experimentally validated human and mouse miRNA targets were retrieved from miRTarBase [12], which manually collected miRNA-target interactions with experimental evidence from the literature. Second, the computationally predicted human and mouse miRNA targets were retrieved from four most widely used miRNA target prediction databases (TargetScan [10], miRDB [13], microRNA.org [14] and DIANA-microT [15]). The miRNA-target interactions in these four databases were predicted by TargetScan algorithm, MirTarget algorithm, miRanda algorithm and DIANA microT-CDS algorithm, respectively. Since the collected miRNA-target interactions from different databases may use different identifiers (IDs), we have to do ID conversion in order to integrate data from different databases. In CSmiRTar, we used miRBase ID as the miRNA identifier and NCBI gene ID as the gene identifier. That is, all miRNA-target interactions were recorded as miRBase ID-NCBI gene ID pairs in CSmiRTar. Third, the tissues in which a human or a mouse gene is expressed were retrieved from Expression Atlas [27]. Expression Atlas, maintained by EMBL-EBI, provided the genes expressed in a specific tissue by analysing microarray and RNA-seq data in ArrayExpress [28]. Fourth, the diseases to which a human gene is related were retrieved from DisGeNET [29], which manually collected gene-disease associations from the literature and other expert curated databases. Fifth, the diseases to which a human miRNA is related were retrieved from PhenomiR [30], which manually collected miRNA-disease associations from the literature. The statistics of CSmiRTar could be found in Table 1. The collected dataset is already very big. On average, a human gene has 572 predicted miRNAs and a mouse gene has 231 predicted miRNAs. Therefore, biologists already have troubles to find out the functional miRNAs (among so many predicted miRNAs) for a gene of interest.

**Table 1:** The statistics of CSmirTar.

Organism	# of collected miRNA-target pairs	# of miRNAs which can be queried	# of genes which can be queried	# of collected tissues in which a gene may be expressed	# of collected diseases to which a gene may be related	# of collected diseases to which a miRNA may be related
Human	19,006,454	2588	21137	81	3696	81
Mouse	6,949,317	1945	21049	51	X	X

### Implementation of CSmiRTar website

CSmiRTar was built using the scripting language PHP and Codelgniter framework. Crawler was used to retrieve raw data from other databases and Python was used to process the raw data. The processed data was stored in MySQL. The Interactive bar chart was generated by Highcharts.

## Utility and discussion

### Database interface

**Fig 1:**
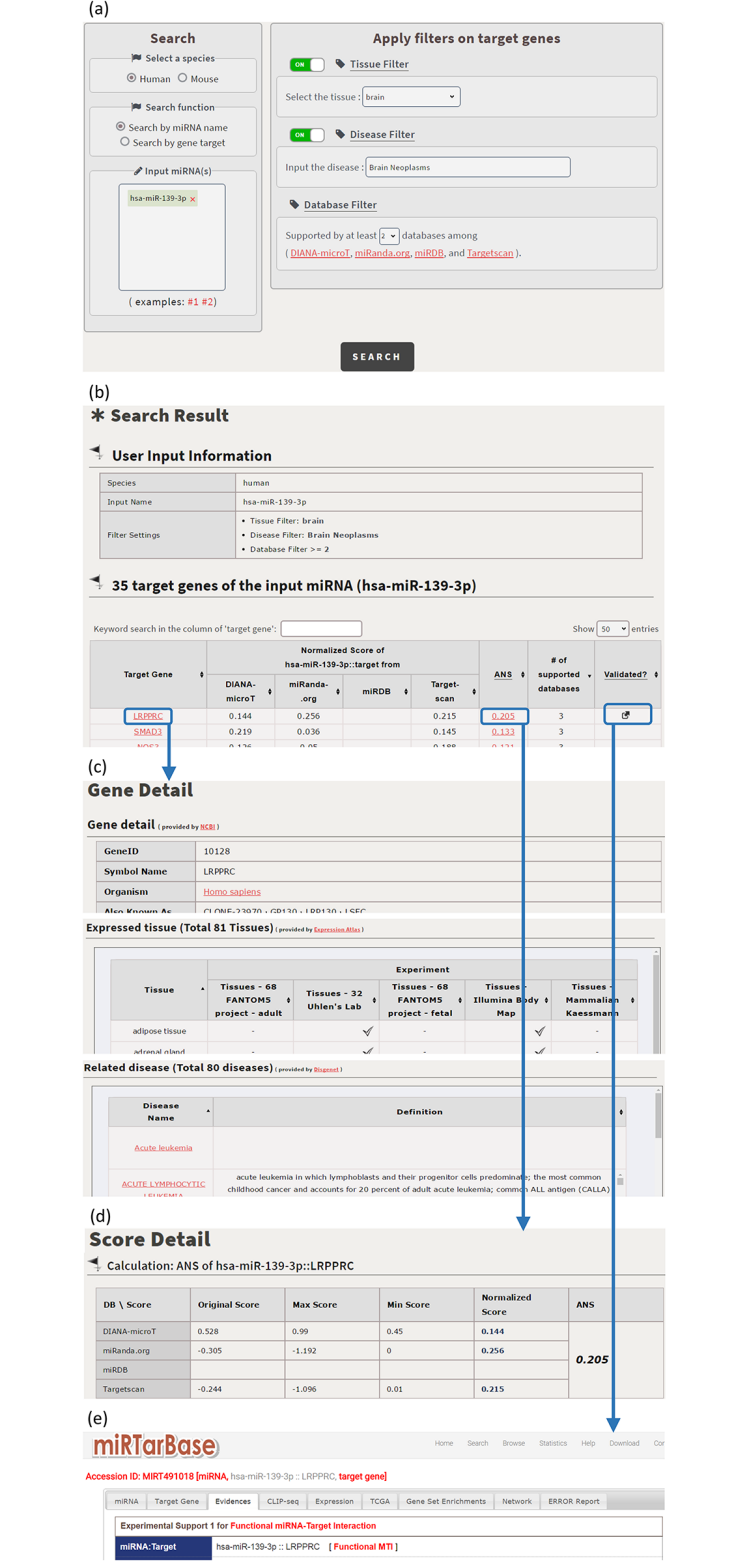
Search the target genes of an input miRNA. (a) Search human miR-139-3p’s target genes which are expressed in the brain tissue, related to the brain neoplasms disease and supported by at least two existing databases. (b) CSmirTar returns 35 target genes sorted by the average normalized score (ANS). (c) When clicking on a gene name in the “Target Gene” column, it opens a webpage showing the basic information of this gene, the tissues in which this gene is expressed and the diseases to which this gene is related. (d) When clicking on a score in the “ANS” column, it opens a webpage showing how the ANS is calculated. (e) When clicking on the icon in the “Validated?” column, it links to miRTarBase to show the experimental evidence of the selected miRNA-target pair.

**Fig 2:**
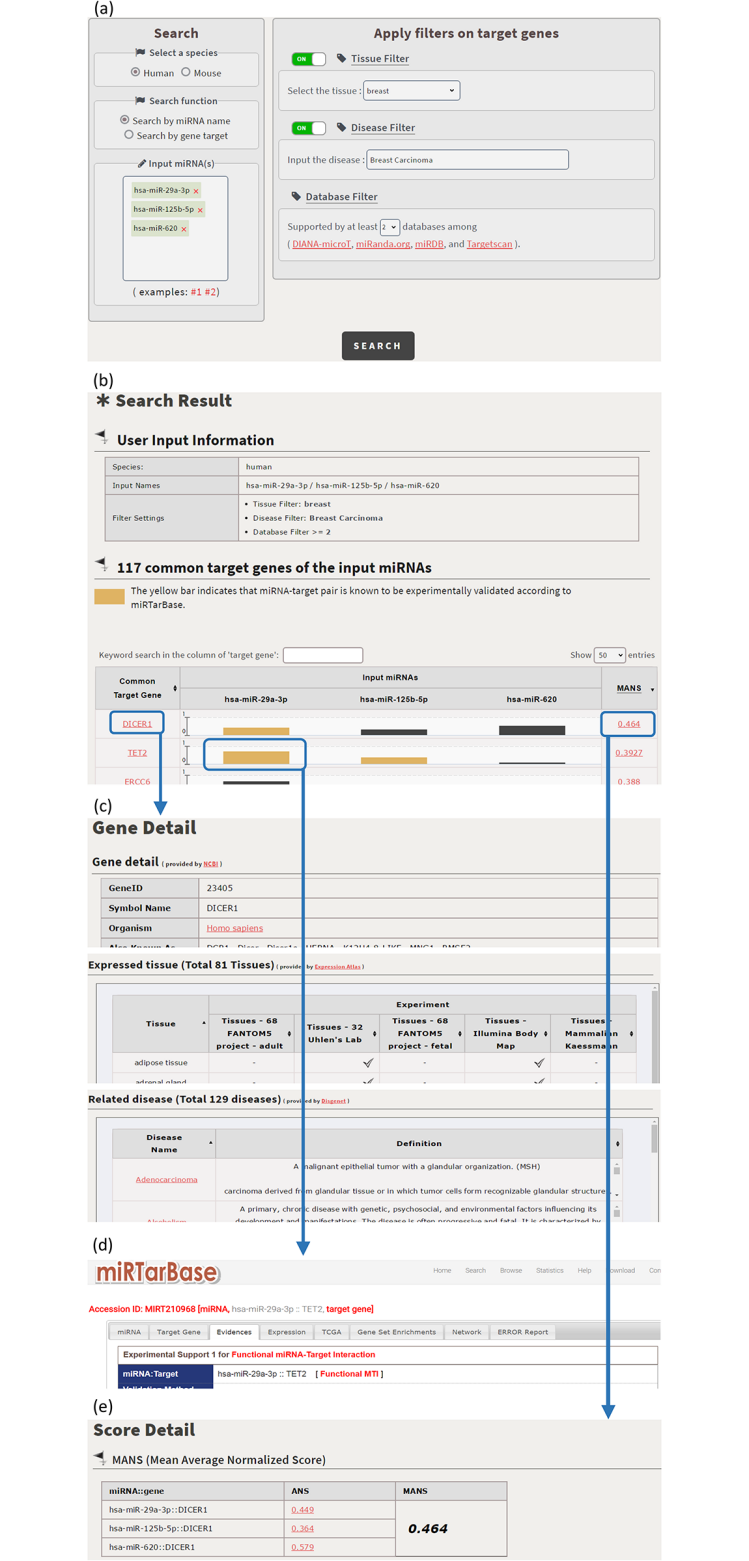
Search the common targets of a set of input miRNAs. (a) For a set of human miRNAs (miR-29a-3p, miR-125b-5p and miR-620), search their common target genes which are expressed in the breast tissue, related to the breast carcinoma disease and supported by at least two existing databases. (b) CSmirTar returns 117 common target genes sorted by the mean average normalized score (MANS). (c) When clicking on a gene name in the “Common Target Gene” column, it opens a webpage showing the basic information of this gene, the tissues in which this gene is expressed and the diseases to which this gene is related. (d) An orange bar means that the miRNA-target pair has been experimentally validated. When clicking on the orange bar, it links to miRTarBase to show the experimental evidence of the selected miRNA-target pair. (e) When clicking on a score in the “MANS” column, it opens a webpage showing how the MANS is calculated.

**Fig 3:**
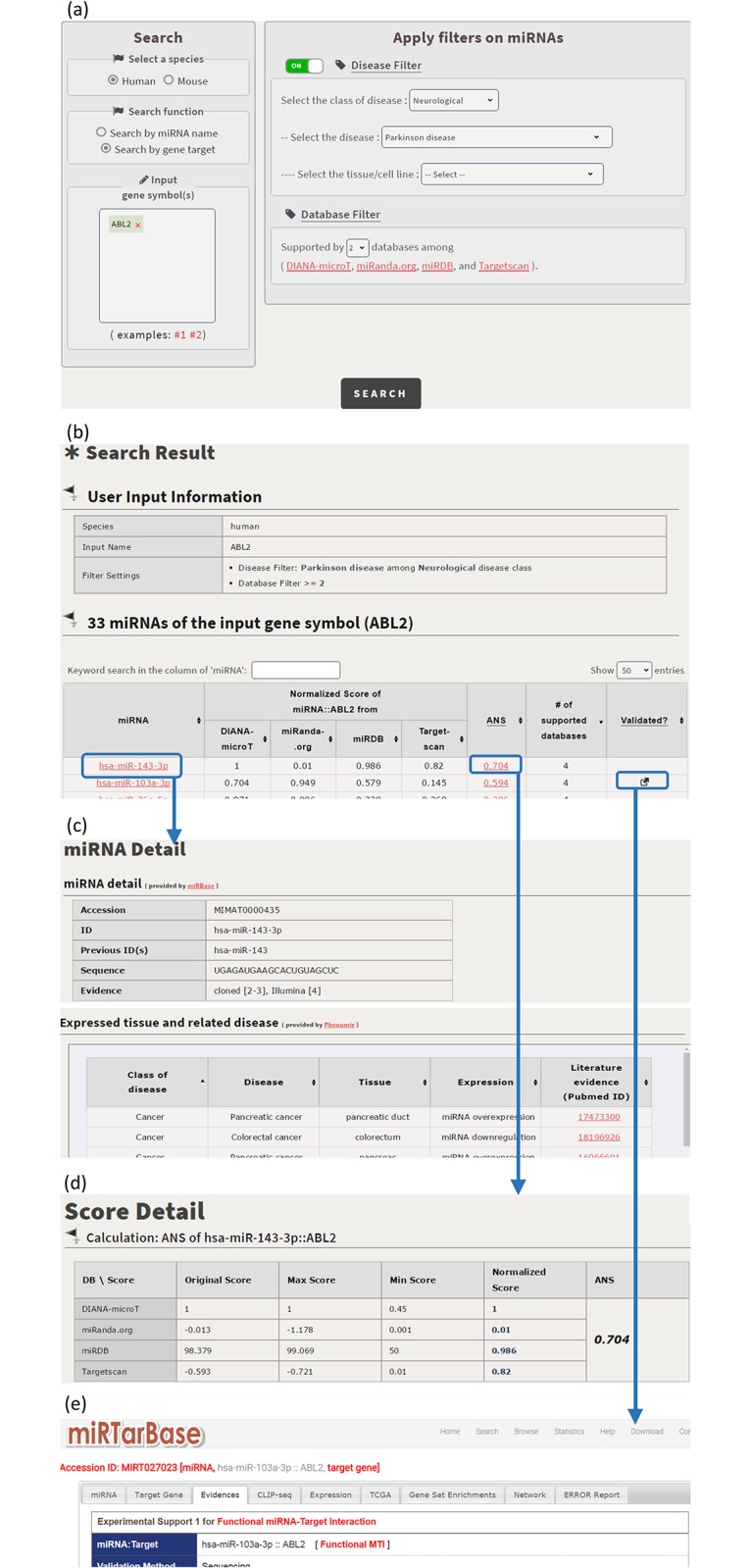
Search the miRNAs of an input gene. (a) For human gene ABL2, search its miRNAs which are related to Parkinson disease and supported by at least two existing databases. (b) CSmirTar returns 33 miRNAs sorted by the average normalized score (ANS). (c) When clicking on a miRNA name in the “miRNA” column, it opens a webpage showing the basic information of this miRNA and the diseases to which this miRNA is related. (d) When clicking on a score in the “ANS” column, it opens a webpage showing how the ANS is calculated. (e) When clicking on the icon in the “Validated?” column, it links to miRTarBase to show the experimental evidence of the selected miRNA-target pair.

**Fig 4:**
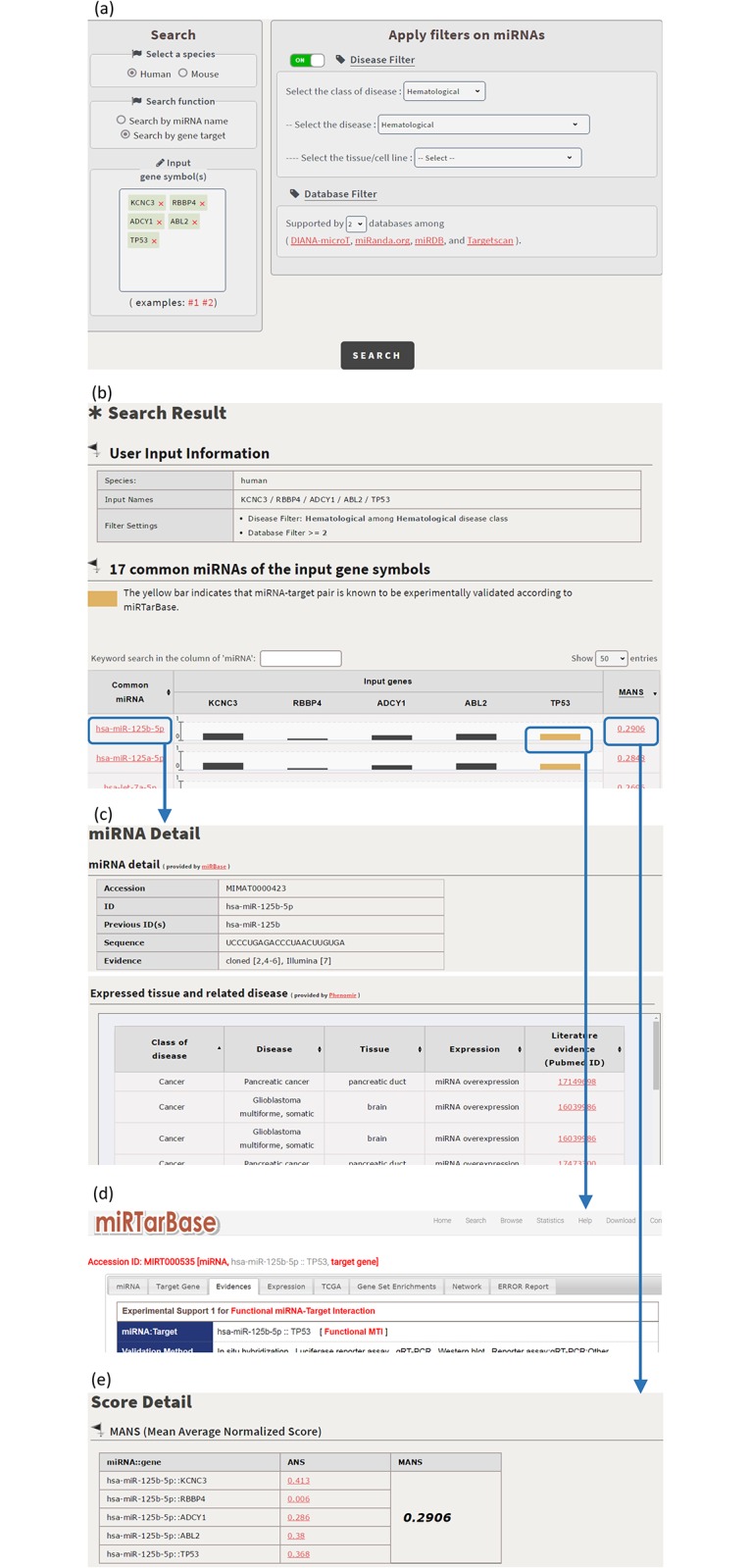
Search the common miRNAs of a set of input genes. (a) For a set of human genes (KCNC3, RBBP4, ADCY1, ABL2 and TP53), search their common miRNAs which are related to the hematological disease and supported by at least two existing databases. (b) CSmirTar returns 17 common miRNAs sorted by the mean average normalized score (MANS). (c) When clicking on a miRNA name in the “Common miRNA” column, it opens a webpage showing the basic information of this miRNA and the diseases to which this miRNA is related. (d) An orange bar means that the miRNA-target pair has been experimentally validated. When clicking on the orange bar, it links to miRTarBase to show the experimental evidence of the selected miRNA-target pair. (e) When clicking on a score in the “MANS” column, it opens a webpage showing how the MANS is calculated.

CSmiRTar provides both a search mode and a browse mode. In the search mode, users have four possible ways to search CSmiRTar. First, users can input a miRNA and search its targets which are (i) expressed in a specific tissue, (ii) related to a specific disease, or/and (iii) supported by multiple existing miRNA target prediction databases. After submission, users will see the search results sorted by the number of supported databases or the average normalized score (see Fig 1). Second, users can input a set of miRNAs and search their common targets which are (i) expressed in a specific tissue, (ii) related to a specific disease, or/and (iii) supported by multiple existing miRNA target prediction databases. After submission, users will see the search results sorted by the number of supported databases or the mean average normalized score (see Fig 2). Third, users can input a gene and search its miRNAs which are (i) related to a specific disease or/and (ii) supported by multiple existing miRNA target prediction databases. After submission, users will see the search results sorted by the number of supported databases or the average normalized score (see Fig 3). Fourth, users can input a set of genes and search their common miRNAs which are (i) related to a specific disease or/and (ii) supported by multiple existing miRNA target prediction databases. After submission, users will see the search results sorted by the number of supported databases or the mean average normalized score (see Fig 4).

**Fig 5:**
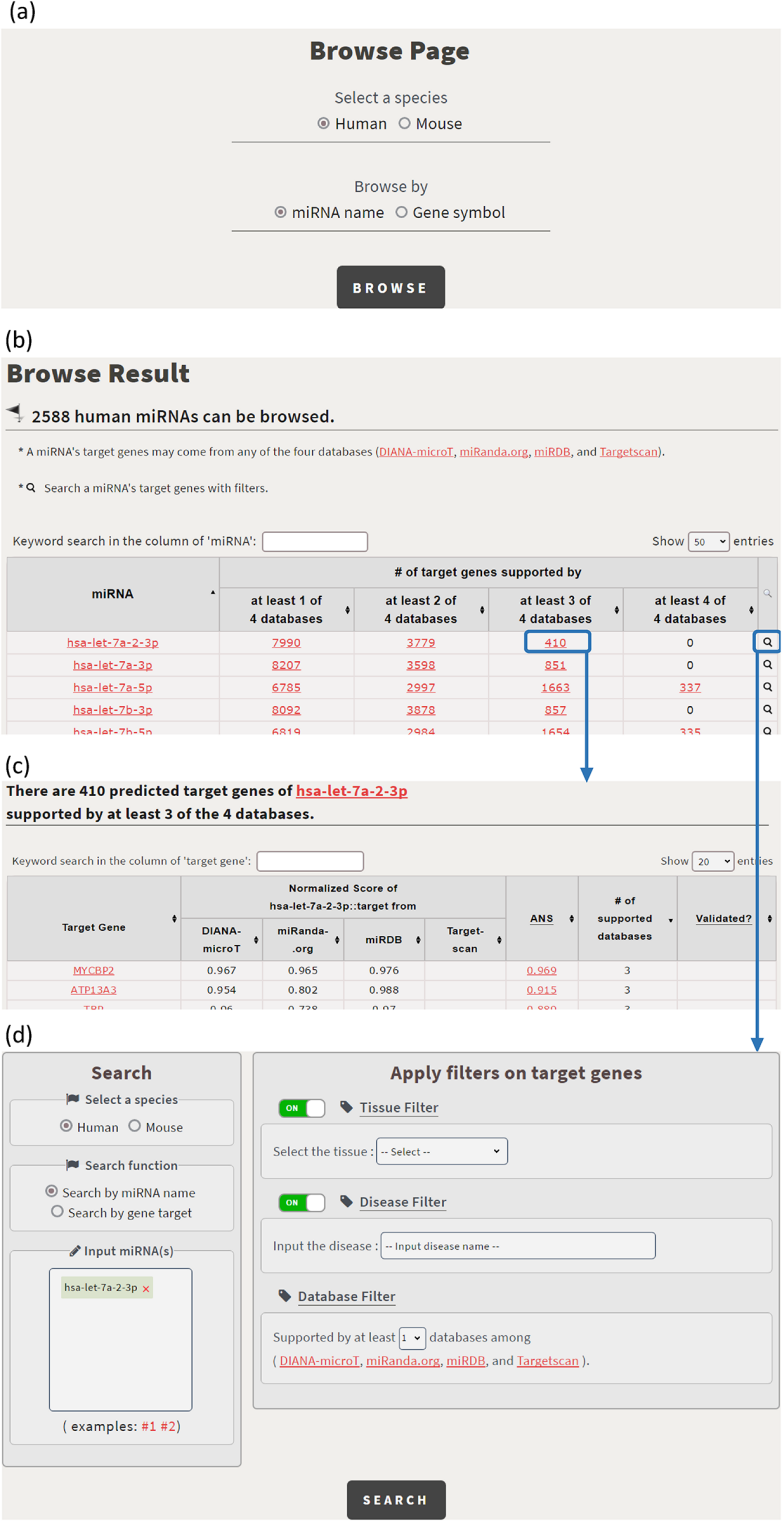
Browse CSmiRTar by a miRNA name. (a) The browse page in shown. (b) The 2588 human miRNAs which can be browsed are shown. (c) By clicking on the number 410, it opens a webpage showing the 410 predicted target genes of hsa-let-7a-2-3p supported by at least 3 of the 4 existing miRNA target prediction databases. (d) By clicking on the icon in the rightmost column, it directs users to the search page.

**Fig 6:**
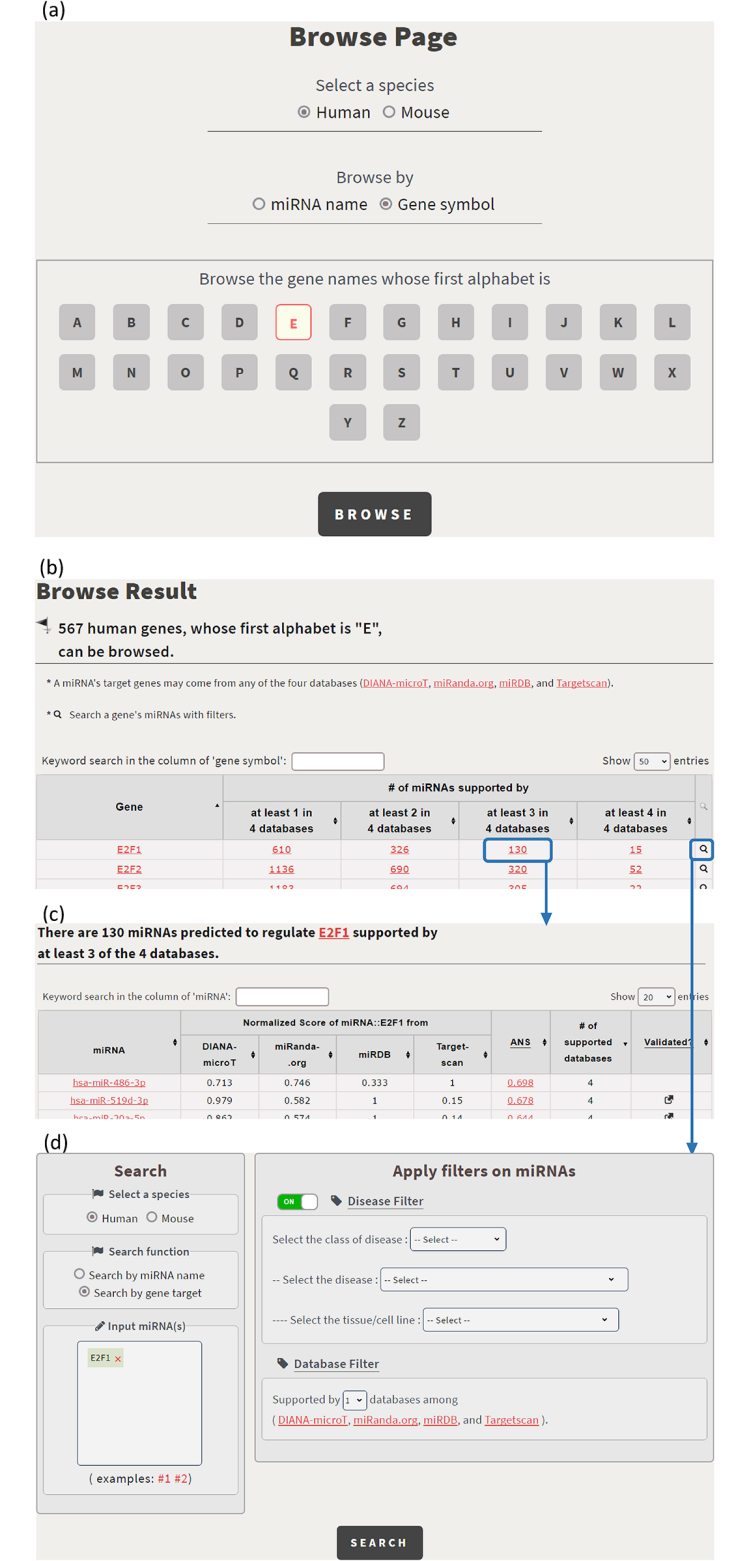
Browse CSmiRTar by a gene name. (a) The browse page is shown. (b) The 567 human genes whose first alphabet is “E” are shown. (c) By clicking on the number 130, it opens a webpage showing the 130 miRNAs predicted to regulate E2F1 supported by at least 3 of the 4 existing miRNA target prediction databases. (d) By clicking on the icon in the rightmost column, it directs users to the search page.

In the browse mode, users have two possible ways to browse CSmiRTar. First, users can click on a human/mouse miRNA name and get the miRNA’s targets supported by one or multiple existing miRNA target prediction databases (see Fig 5). Second, users can click on a human/mouse gene name and get the miRNAs, which regulate the gene, supported by one or multiple existing miRNA target prediction databases (see Fig 6).

### Our database/tissue/disease filters can significantly reduce the number of predicted miRNA targets but still keep the functional ones

Identifying the functional targets is a crucial step to dissect the function of miRNAs. Using existing miRNA target prediction databases usually returns thousands of predicted targets per miRNA. Therefore, it is very hard for researchers to choose the biologically plausible candidates for further experimental validation. Besides, it can be expected that many of the predicted targets are non-functional since miRNAs only regulate their target genes in specific tissues, cell types and disease states.

To solve this problem, we implement three different kinds of filters (a database filter, a tissue filter and a disease filter) to efficiently reduce the number of predicted miRNA targets but still keep the functional ones. To show the effectiveness of our filters, we prepare a benchmark set by randomly selecting several experimentally validated miRNA-target pairs in specific tissues and cancers from OncomiRDB [31]. As shown in Table 2 for 10 case studies, our filters can significantly reduce the number of predicted miRNA targets by more than 90% but still keep the experimentally validated miRNA targets. For example, human miR-16-5p is known to regulate the gene PPM1D in breast cancer cells [32]. Even by considering the common predicted targets from four existing miRNA target prediction databases (i.e. setting the database filter equal to four), PPM1D is still hidden in 883 predicted targets of miR-16-5p, suggesting that applying the database filter alone is not an efficient way to reduce the non-functional miRNA targets. If we further apply the tissue filter (selecting breast) and disease filter (selecting invasive breast cancer), PPM1D is now hidden in only 16 predicted targets. Researchers then have a high chance to pick out the functional targets (e.g. PPM1D) of miR-16-5p for further experimental investigation. On the contrary, if using existing miRNA target prediction databases (e.g. miRecords [16], miRWalk [22], miRSystem [19] and starBase [23]), researchers will have difficulty to pick out PPM1D among hundreds or even thousands of predicted targets of miR-16-5p (see Table 3).

**Table 2:** Our database/tissue/disease filters can significantly reduce the number of predicted miRNA targets but still keep the functional one.

Human miRNA	Functional target	Reference Pubmed ID	Filter settings: (database/tissue/disease)	# of predicted targets when using only the database filter	# of predicted targets when using all three (database/tissue/disease) filters	Reduced ratio
miR-16-5p	PPM1D	20668064	4 / breast tissue / invasive breast cancer	883	16	98.19% = (883–16)/883
miR-301a-3p	PTEN	21393507	4 / breast tissue / invasive breast cancer	533	14	97.37%
miR-195-5p	SLC2A3	22265971	3 / bladder tissue / carcinoma of bladder	3008	125	95.84%
miR-615-5p	IGF2	22819824	3 / liver tissue / liver neoplasms	1238	57	95.40%
miR-519d-3p	CDKN1A	22262409	4 / liver tissue / liver neoplasms	717	45	93.72%
miR-135a-5p	APC	18632633	4 / colon / colorectal neoplasms	374	27	92.78%
miR-153-3p	MCL1	19676043	3 / brain / glioblastoma	1816	156	91.41%
miR-204-5p	EZR	21416062	3 / stomach / stomach carcinoma	1152	104	90.97%
miR-103a-3p	KLF4	22593189	3 / colon / colorectal carcinoma	2095	236	88.74%
miR-497-5p	RAF1	21350001	4 / breast tissue / breast neoplasms	887	106	88.05%

**Table 3:** The number of predicted miRNA targets, which include the functional one being examined, in different databases.

Human miRNA	Functional target	miRecords	miRWalk	miRSystem	starBase	CSmiRTar
miR-16-5p	PPM1D	151 (6)^a^	916 (8)	151 (6)	4239 (1)	16
miR-301a-3p	PTEN	352 (5)	715 (8)	561 (4)	144 (4)	14
miR-195-5p	SLC2A3	479 (5)	942 (8)	404 (5)	338 (4)	125
miR-615-5p	IGF2	66 (4)	196 (8)	3036 (1)	N/A	57
miR-519d-3p	CDKN1A	217 (5)	806 (8)	19 (6)	4 (5)	45
miR-135a-5p	APC	687 (4)	1336 (7)	59 (6)	2262 (1)	27
miR-153-3p	MCL1	42 (6)	111 (9)	177 (5)	246 (3)	156
miR-204-5p	EZR	224 (5)	9 (11)	14 (7)	172 (3)	104
miR-103a-3p	KLF4	3799 (3)	1751 (7)	1983 (2)	299 (3)	236
miR-497-5p	RAF1	11043 (2)	1303 (8)	224 (5)	342 (4)	106

miRecords, miRWalk, miRSystem and starBase were all run with the setting of their algorithm filter as stringent as possible to minimize the number of predicted mRNA targets while still include the functional target being examined. CSmiRTar was run with the settings shown in Table 2.

- ^a^151 (6) means that in miRecords, miR-16-5p has 151 target genes (including PPM1D) predicted by at least 6 different algorithms. Note that in miRecords, 6 is the most stringent setting of the algorithm filter for still reporting PPM1D as a predicted target gene of miR-16-5p.

### Our database/disease filters can significantly reduce the number of predicted miRNAs of a gene but still keep the functional ones

As shown in Table 4 for 10 case studies, our filters can significantly reduce the number of predicted miRNAs of a gene by 63% to 95% but still keep the experimentally validated miRNAs which really regulate the gene. For example, human gene MECP2 is known to be regulated by miR-212-3p in gastric cancer cells [33]. Even by considering the common predicted miRNAs from three existing miRNA target prediction databases (i.e. setting the database filter equal to three), miR-212-3p is still hidden in 537 predicted miRNAs of the gene MECP2, suggesting that applying the database filter alone is not an efficient way to reduce the non-functional miRNAs of MECP2. If we further apply the disease filter (selecting gastric cancer in the stomach), miR-212-3p is now hidden in only 22 predicted miRNA of MECP2. Researchers then have a high chance to pick out the functional miRNAs (e.g. miR-212-3p) of MECP2 for further experimental investigation.

**Table 4:** The database/disease filters can significantly reduce the number of predicted miRNAs but still keep the functional miRNA which regulates the target gene.

Human target gene	Functional miRNA which regulates the target gene	Reference Pubmed ID	Filter setting: (database/disease)	# of predicted miRNAs when using only the database filter	# of predicted miRNAs when using both (database/disease) filters	Reduced ratio
MECP2	miR-212-3p	20020497	3 / gastric cancer in stomach	537	22	95.90% = (537–22)/537
MCL1	miR-153-3p	19676043	3 / glioblastoma multiforme, somatic in brain	253	21	91.70%
CREB1	miR-181b-5p	22539488	4 / gastric cancer in stomach	99	9	90.91%
ALCAM	miR-215-5p	21119604	4 / gastric cancer in stomach	47	5	89.36%
FOXO1	miR-370-3p	23029264	4 / prostate cancer in prostate gland	49	7	85.71%
APC	miR-135a-5p	18632633	4 / colorectal cancer in colorectum	22	5	77.27%
SPRY2	miR-27a-3p	20638779	4 / pancreatic cancer in pancreas	26	6	76.92%
KLF4	miR-103a-3p	22593189	3 / colorectal cancer in colorectum	128	35	72.66%
ID1	miR-381-3p	22592211	3 / lung cancer in lung cancer cell line	56	16	71.43%
PBX3	let-7c-5p	21984339	4 / colorectal cancer in colorectum	60	22	63.33%

### Identifying the shared miRNAs of ceRNAs

An important step to reconstruct the ceRNA network is to identify the shared miRNAs of ceRNAs. CSmiRTar allows users to input a set of genes (e.g. ceRNAs) to search the shared miRNAs which regulate these genes. As shown in Table 5 for five case studies, CSmiRTar can identify the experimentally validated shared miRNAs of ceRNAs. For example, it is known that human ceRNAs (PTEN, VAPA and CNOT6L) are all regulated by miR-17-5p, miR-19a-3p, miR-20a-5p and miR-106b-5p in human prostate cancer cells [34]. By considering the common predicted miRNAs from three existing databases (i.e. setting the database filter equal to three) and applying the disease filter (selecting prostate cancer), CSmiRTar returns 13 predicted shared miRNAs which contain all the four experimentally validated shared miRNAs of the input ceRNAs.

**Table 5:** The database/disease filters can significantly reduce the number of predicted shared miRNAs but still keep the functional shared miRNAs of ceRNAs.

Human ceRNAs	Functional shared miRNAs	Reference Pubmed ID	Filter setting: (database/disease)	# of predicted shared miRNAs when using the filters
PTEN, VAPA, CNOT6L	miR-17-5p, miR-19a-3p, miR-20a-5p, miR-106b-5p	22000013	3 / prostate cancer	13
CD44, CDC42	miR-216a-5p, miR-330-3p	21149267	1 / breast cancer	192
PTENP1, PTEN	miR-19b-3p, miR-20a-5p	20577206	1 / colorectal cancer	114
CDK6, ABL1, SRC	miR-203-3p	23462723	2 / breast cancer	29
FOXF2, RECK, MTSS1	miR-182-5p	23383207	3 / prostate cancer	2

### Identifying the common target genes of a set of miRNAs

In CSmiRTar, users can input a set of miRNAs to search their common target genes. As shown in Table 6 for five case studies, CSmiRTar can successfully identify the experimentally validated common target genes of multiple miRNAs. For example, it is known that human miR-186-5p, miR-216b-5p, miR-337-3p, and miR-760 cooperatively induce cellular senescence by targeting the gene CSNK2A1 in human colorectal cancer cells [35]. By considering the predicted target genes supported by three existing miRNA target prediction databases (i.e. setting the database filter equal to three) and applying the tissue/disease filter (selecting colon/colorectal carcinoma), CSmiRTar returns 23 predicted common target genes which contain the experimentally validated common target gene CSNK2A1 of the input set of miRNAs.

**Table 6:** The database/tissue/disease filters can significantly reduce the number of predicted common targets but still keep the functional common target of a set of miRNAs.

A set of human miRNAs	Common target gene	Reference Pubmed ID	Filter settings: (database/tissue/disease)	# of predicted targets when using the filters
miR-17-5p, miR-19a-3p, miR-20a-5p, miR-20b-5p, miR-26b-5p, miR-106a-5p, miR-106b-5p	PTEN	22000013	3 / prostate tissue / prostate carcinoma	20
miR-186-5p, miR-216b-5p, miR-337-3p, miR-760	CSNAK2A1	23137536	3 / colon / colorectal carcinoma	23
miR-330-3p, miR-608, miR-216a-5p	CD44	21149267	2 / breast tissue / breast neoplasms	78
miR-130a-3p, miR-301a-3p, miR-454-3p	SMAD4	23393589	3 / colon / colorectal neoplasms	116
miR-19b-3p, miR-20a-5p	PTEN	20577206	3 / colon tissue / colon carcinoma	91

## Conclusions

In this article, we present CSmiRTar which provide computationally predicted targets of 2588 human miRNAs and 1945 mouse miRNAs. CSmiRTar implements (i) a tissue filter for users to search the miRNA targets expressed in a specific tissue, (ii) a disease filter for users to search the miRNA targets related to a specific disease, and (iii) a database filter for users to search the predicted miRNA targets supported by multiple existing databases,. Moreover, CSmiRTar allows users to search the common targets of a set of input miRNAs under a specific physiological condition and the common miRNAs of a set of input genes under a specific physiological condition. We provide many case studies to show the effectiveness of our filters in reducing the number of predicted miRNA targets but still keep the functional ones. However, users should note that some functional miRNA targets may not be kept when applying both the tissue and disease filters if they are not expressed in normal tissues but are abnormally expressed in disease states. Nevertheless, we believe that CSmiRTar will be a useful database for biologists to study the molecular mechanisms of post-transcriptional regulation in human and mouse.

## References

[1] Bartel DP. MicroRNAs: genomics, biogenesis, mechanism, and function. Cell 2004; 116(2):281–297. 14744438 10.1016/s0092-8674(04)00045-5

[2] Filipowicz W, Jaskiewicz L, Kolb FA, Pillai RS. Post-transcriptional gene silencing by siRNAs and miRNAs. Curr Opin Struct Biol 2005; 15(3):331–341. doi: 10.1016/j.sbi.2005.05.006 15925505 10.1016/j.sbi.2005.05.006

[3] Sontheimer EJ, Carthew RW. Silence from within: endogenous siRNAs and miRNAs. Cell 2005; 122(1):9–12. doi: 10.1016/j.cell.2005.06.030 16009127 10.1016/j.cell.2005.06.030

[4] Lewis BP, Burge CB, Bartel DP. Conserved seed pairing, often flanked by adenosines, indicates that thousands of human genes are microRNA targets. Cell 2005; 120(1):15–20. doi: 10.1016/j.cell.2004.12.035 15652477 10.1016/j.cell.2004.12.035

[5] Bartel DP. MicroRNAs: target recognition and regulatory functions. Cell 2009; 136(2):215–233. doi: 10.1016/j.cell.2009.01.002 19167326 10.1016/j.cell.2009.01.002 PMC3794896

[6] Huntzinger E, Izaurralde E. Gene silencing by microRNAs: contributions of translational repression and mRNA decay. Nat Rev Genet 2011, 12(2):99–110. doi: 10.1038/nrg2936 21245828 10.1038/nrg2936

[7] Krutzfeldt J, Rajewsky N, Braich R, Rajeev KG, Tuschl T, Manoharan M, et al Silencing of microRNAs in vivo with ‘antagomirs’. Nature 2005; 438(7068):685–689. doi: 10.1038/nature04303 16258535 10.1038/nature04303

[8] Bushati N, Cohen SM. MicroRNA functions. Annu Rev Cell Dev Biol 2007; 23:175–205. doi: 10.1146/annurev.cellbio.23.090506.123406 17506695 10.1146/annurev.cellbio.23.090506.123406

[9] Stefani G, Slack FJ. Small non-coding RNAs in animal development. Nat Rev Mol Cell Biol 2008; 9(3):219–230. doi: 10.1038/nrm2347 18270516 10.1038/nrm2347

[10] Agarwal V, Bell GW, Nam JW, Bartel DP. Predicting effective microRNA target sites in mammalian mRNAs. Elife 2015; 4:e05005. 10.7554/eLife.05005 PMC4532895 26267216

[11] Vlachos IS, Paraskevopoulou MD, Karagkouni D, Georgakilas G, Vergoulis T, Kanellos I, et al DIANA-TarBase v7.0: indexing more than half a million experimentally supported miRNA:mRNA interactions. Nucleic Acids Res 2015; 43:D153–D159. doi: 10.1093/nar/gku1215 25416803 10.1093/nar/gku1215 PMC4383989

[12] Hsu SD, Tseng YT, Shrestha S, Lin YL, Khaleel A, Chou CH, et al miRTarBase update 2014: an information resource for experimentally validated miRNA-target interactions. Nucleic Acids Res 2014; 42:D78–D85. doi: 10.1093/nar/gkt1266 24304892 10.1093/nar/gkt1266 PMC3965058

[13] Wong N, Wang X. miRDB: an online resource for microRNA target prediction and functional annotations. Nucleic Acids Res 2015; 43:D146–D152. doi: 10.1093/nar/gku1104 25378301 10.1093/nar/gku1104 PMC4383922

[14] Betel D, Wilson M, Gabow A, Marks DS, Sander C. The microRNA.org resource: targets and expression. Nucleic Acids Res 2008; 36:D149–D153. doi: 10.1093/nar/gkm995 18158296 10.1093/nar/gkm995 PMC2238905

[15] Paraskevopoulou MD, Georgakilas G, Kostoulas N, Vlachos IS, Vergoulis T, Reczko M, et al DIANA-microT web server v5.0: service integration into miRNA functional analysis workflows. Nucleic Acids Res 2013; 41:W169–W173. doi: 10.1093/nar/gkt393 23680784 10.1093/nar/gkt393 PMC3692048

[16] Xiao F, Zuo Z, Cai G, Kang S, Gao X, Li T. miRecords: an integrated resource for microRNA-target interactions. Nucleic Acids Res 2009; 37:D105–D110. doi: 10.1093/nar/gkn851 18996891 10.1093/nar/gkn851 PMC2686554

[17] Sales G, Coppe A, Bisognin A, Biasiolo M, Bortoluzzi S, Romualdi C. MAGIA, a web-based tool for miRNA and genes integrated analysis. Nucleic Acids Res 2010; 38:W352–W359. doi: 10.1093/nar/gkq423 20484379 10.1093/nar/gkq423 PMC2896126

[18] Shirdel EA, Xie W, Mak TW. NAViGaTing the micronome—using multiple microRNA prediction databases to identify signalling pathway-associated microRNAs. PLoS One 2011; 6(2):e17429 doi: 10.1371/journal.pone.0017429 21364759 10.1371/journal.pone.0017429 PMC3045450

[19] Lu TP, Lee CY, Tsai MH, Chiu YC, Hsiao CK, Lai LC, et al miRSystem: an integrated system for characterizing enriched functions and pathways of microRNA targets. PLoS One 2012; 7(8):e42390 doi: 10.1371/journal.pone.0042390 22870325 10.1371/journal.pone.0042390 PMC3411648

[20] Cho S, Jang I, Jun Y. MiRGator v3.0: a microRNA portal for deep sequencing, expression profiling and mRNA targeting. Nucleic Acids Res 2013; 41:D252–D257. doi: 10.1093/nar/gks1168 23193297 10.1093/nar/gks1168 PMC3531224

[21] Preusse M, Theis FJ, Mueller NS. miTALOS v2: analyzing tissue specific microRNA function. PLoS One 2016; 11(3):e0151771 doi: 10.1371/journal.pone.0151771 26998997 10.1371/journal.pone.0151771 PMC4801359

[22] Dweep H, Gretz N. miRWalk2.0: a comprehensive atlas of microRNA-target interactions. Nat Methods 2015; 12(8):697 doi: 10.1038/nmeth.3485 26226356 10.1038/nmeth.3485

[23] Li JH, Liu S, Zhou H, Qu LH, Yang JH. starBase v2.0: decoding miRNA-ceRNA, miRNA-ncRNA and protein-RNA interaction networks from large-scale CLIP-Seq data. Nucleic Acids Res 2014; 42:D92–D97. doi: 10.1093/nar/gkt1248 24297251 10.1093/nar/gkt1248 PMC3964941

[24] Ebert MS, Neilson JR, Sharp PA. MicroRNA sponges: competitive inhibitors of small RNAs in mammalian cells. Nat Methods 2007, 4(9):721–726. doi: 10.1038/nmeth1079 17694064 10.1038/nmeth1079 PMC3857099

[25] Karreth FA, Tay Y, Perna D, Ala U, Tan SM, Rust AG, et al In vivo identification of tumor-suppressive PTEN ceRNAs in an oncogenic BRAF-induced mouse model of melanoma. Cell 2011; 147(2):382–395. doi: 10.1016/j.cell.2011.09.032 22000016 10.1016/j.cell.2011.09.032 PMC3236086

[26] Qi X, Zhang DH, Wu N, Xiao JH, Wang X, Ma W. ceRNA in cancer: possible functions and clinical implications. J Med Genet 2015; 52(10):710–718. doi: 10.1136/jmedgenet-2015-103334 26358722 10.1136/jmedgenet-2015-103334

[27] Petryszak R, Burdett T, Fiorelli B, Fonseca NA, Gonzalez-Porta M, Hastings E, et al Expression Atlas update—a database of gene and transcript expression from microarray-and sequencing-based functional genomics experiments. Nucleic Acids Res 2014; 42:D926–D932. doi: 10.1093/nar/gkt1270 24304889 10.1093/nar/gkt1270 PMC3964963

[28] Kolesnikov N, Hastings E, Keays M, Melnichuk O, Tang YA, Williams E, et al ArrayExpress update—simplifying data submissions. Nucleic Acids Res 2015; 43(Database issue):D1113–D1116. doi: 10.1093/nar/gku1057 25361974 10.1093/nar/gku1057 PMC4383899

[29] Piñero J, Queralt-Rosinach N, Bravo À, Deu-Pons J, Bauer-Mehren A, Baron M, et al DisGeNET: a discovery platform for the dynamical exploration of human diseases and their genes. Database 2015; bav028 doi: 10.1093/database/bav028 25877637 10.1093/database/bav028 PMC4397996

[30] Ruepp A, Kowarsch A, Schmidl D, Buggenthin F, Brauner B, Dunger I, et al PhenomiR: a knowledgebase for microRNA expression in diseases and biological processes. Genome Biol 2010, 11(1):R6 doi: 10.1186/gb-2010-11-1-r6 20089154 10.1186/gb-2010-11-1-r6 PMC2847718

[31] Wang D, Gu J, Wang T, Ding Z. OncomiRDB: a database for the experimentally verified oncogenic and tumor-suppressive microRNAs. Bioinformatics 2014; 30(15):2237–2238. doi: 10.1093/bioinformatics/btu155 24651967 10.1093/bioinformatics/btu155

[32] Zhang X, Wan G, Mlotshwa S, Vance V, Berger FG, Chen H, et al Oncogenic Wip1 phosphatase is inhibited by miR-16 in the DNA damage signaling pathway. Cancer Res 2010, 70(18):7176–7186. doi: 10.1158/0008-5472.CAN-10-0697 20668064 10.1158/0008-5472.CAN-10-0697 PMC2940956

[33] Wada R, Akiyama Y, Hashimoto Y, Fukamachi H, Yuasa Y. miR-212 is downregulated and suppresses methyl-CpG-binding protein MeCP2 in human gastric cancer. Int J Cancer 2010; 127(5):1106–1114. doi: 10.1002/ijc.25126 20020497 10.1002/ijc.25126

[34] Tay Y, Kats L, Salmena L, Weiss D, Tan SM, Ala U, et al Coding-independent regulation of the tumor suppressor PTEN by competing endogenous mRNAs. Cell 2011; 147(2):344–357. doi: 10.1016/j.cell.2011.09.029 22000013 10.1016/j.cell.2011.09.029 PMC3235920

[35] Kim SY, Lee YH, Bae YS. MiR-186, miR-216b, miR-337-3p, and miR-760 cooperatively induce cellular senescence by targeting α subunit of protein kinase CKII in human colorectal cancer cells. Biochem Biophys Res Commun 2012; 429(3–4):173–179. doi: 10.1016/j.bbrc.2012.10.117 23137536 10.1016/j.bbrc.2012.10.117

